# Pioglitazone inhibits high glucose-induced expression of receptor for advanced glycation end products in coronary artery smooth muscle cells

**DOI:** 10.3892/mmr.2014.3113

**Published:** 2014-12-18

**Authors:** BEI-BING DI, HONG-WEI LI, WEI-PING LI, XU-HUA SHEN, ZHI-JUN SUN, XING WU

**Affiliations:** Department of Cardiology, Beijing Friendship Hospital, Capital Medical University, Beijing 100050, P.R. China

**Keywords:** receptor for advanced glycation end products, pioglitazone, reactive oxygen species, nuclear factor-κB, peroxisome proliferator-activated receptor γ

## Abstract

Receptor for advanced glycation end products (RAGE) is critical in inflammatory diseases, including diabetes and atherosclerosis. The mechanism underlying the effect of peroxisome proliferator-activated receptor γ (PPARγ) agonist pioglitazone (PIO) on RAGE expression in coronary artery smooth muscle cells (SMCs) stimulated by high glucose concentrations remains to be elucidated. In the present study, the effect and mechanism of action of PIO on RAGE expression in SMCs was investigated following treatment with high glucose concentrations. Rat coronary artery SMCs were pretreated with PIO alone, PIO and GW9662 (a PPARγ antagonist), diphenyleneiodonium (DPI; a nicotinamide adenine dinucleotide phosphate (NADPH) oxidase inhibitor) or the antioxidant pyrrolidine dithiocarbamate (PDTC; a nuclear factor-κB (NF-κB) inhibitor), followed by treatment with high glucose. RAGE mRNA and protein expression, reactive oxygen species (ROS) production and NF-κB nuclear translocation were investigated. Glucose induced RAGE expression in a dose-dependent manner, with maximal effect at a concentration of 25 mmol/l following treatment for 48 h. PIO, DPI and PDTC reduced high glucose-induced increases in RAGE protein and mRNA expression. PIO prominently downregulated RAGE expression and inhibited high glucose-induced increases in ROS production and NF-κB activation (P<0.05). Pretreatment with PIO and GW9662 did not exhibit this inhibitory effect. High glucose may stimulate RAGE expression in coronary artery SMCs through NADPH oxidase-mediated ROS generation and NF-κB activation. PIO downregulated RAGE expression and inhibited ROS production and NF-κB activation via PPARγ activation, which may prevent the inflammatory effect of AGE/RAGE system in diabetes.

## Introduction

The receptor for advanced glycation end products (RAGE) is a multiligand receptor of the immunoglobulin (Ig) superfamily of cell surface molecules, first introduced as a critical factor in diabetes and other metabolic disorders characterized by AGE accumulation. As well as binding with AGEs, RAGE interacts with high-mobility group box protein 1, members of the S100/calgranulin family, β2-integrin Mac, amyloid-β peptide and β-sheet fibrils (1–3). A previous *in vivo* study revealed that RAGE mRNA and protein expression significantly increased in the coronary arteries of type II diabetic mice compared with non-diabetic mice (4). AGE and RAGE expression were upregulated at sites of endothelial denudation. This was particularly prevalent in activated smooth muscle cells (SMCs) of the expanding neointima in mice (5). SMC proliferation, migration and neointimal expansion upon arterial injury were markedly suppressed in homozygous RAGE null mice compared with wildtype litter mates (5). Furthermore, RAGE overexpression was associated with enhanced inflammatory reactions and increased expression of cyclooxygenase-2, microsomal prostaglandin synthase-1, matrix metalloproteinase-2 (MMP-2) and MMP-9 in plaque macrophages and SMCs in diabetic patients (6).

The nuclear receptor peroxisome proliferator-activated receptor γ (PPARγ) is a member of the nuclear hormone receptor superfamily of ligand-activated transcriptional factors. Previously, PPARγ activators were demonstrated to exert anti-inflammatory, anti-oxidative and anti-proliferative effects on vascular wall cells (7). A number of studies have revealed that PPARγ agonists inhibit the deleterious effects of AGEs in cell culture and animal models. Pretreatment of rat aortic SMCs with PPARγ agonist rosiglitazone, significantly downregulated RAGE expression and subsequently inhibited SMC proliferation in response to treatment with RAGE agonists S100/calgranulin (7). Insulin inhibited AGE-induced SMC proliferation by not only suppressing nuclear factor-κB (NF-κB) activation, but also by increasing PPARγ expression (8). Furthermore, rosiglitazone was reported to decrease RAGE expression and SMC proliferation following carotid arterial injury in diabetic and non-diabetic rats (7).

It remains to be elucidated if high glucose is able to induce coronary artery SMC RAGE expression and the mechanism has not been investigated. In addition, little is known with regard to the effect of pioglitazone on high glucose-induced RAGE expression in coronary artery vascular SMCs (VSMCs), which are the main cell type in coronary atherosclerosis. In the present study, RAGE expression in coronary artery SMCs was investigated following treatment with varying concentrations of glucose. The effects of the PPARγ agonist pioglitazone (PIO) on RAGE expression in VSMCs and its underlying mechanism following treatment with high concentrations of glucose was also investigated.

## Materials and methods

### Materials

Sprague-Dawley (SD) rats were purchased from Vital River Laboratories Animals Technology Co., Ltd. (Beijing, China). PIO was donated by Huadong Medicine Co., Ltd. (Hangzhou, China). GW9662, diphenylene iodonium (DPI) and pyrrolidine dithiocarbamate (PDTC) were purchased from Sigma (St. Louis, MO, USA). Dulbecco’s modified Eagle medium (DMEM) and fetal bovine serum (FBS) were purchased from Hyclone Laboratories, Inc. (Logan, UT, USA). TRIzol^®^ reagent and cell lysates were purchased from Invitrogen Life Technologies (Shanghai, China). Anti-GAPDH was purchased from ProMab Biotechnologies Inc. (Richmond, CA, USA) Anti-RAGE was obtained from Abcam (Hong Kong, SAR, P.R. China). Anti-NF-κB p65, anti-I-κBα, fluorescein isothiocyanate (FITC)-labeled goat anti-rabbit IgG and the reactive oxygen species (ROS) assay kit were purchased from the Beyotime Institute of Biotechnology (Jiangsu, China).

### Cell culture and treatment

Male SD rats (Vital River Laboratories Animals Technology Co., Ltd.) weighing 250 g were housed in an environmentally controlled room with a 12-h light/dark cycle and administered standard rodent chow and tap water *ad libitum*. The present study was approved by the ethics committee of Beijing Friendship Hospital, Capital Medical University (Beijing, China). Male SD rats at seven weeks old were anesthetized with bentobarbital sodium (60 mg/kg). Rat coronary arteries were dissected from the ventricle and the endothelium in the vessels was denuded with air. Enzymatic isolation of VSMCs was performed according to published methods (9). Coronary artery VSMCs were cultured in DMEM supplemented with 15% fetal calf serum, 100 U/ml penicillin-G and 100 mg/ml streptomycin. The morphology and growth characteristics of the cells were typical of SMCs and were identified as SMCs by positive α-smooth muscle actin staining. Cultured cells were incubated in DMEM with 15% FBS, followed by synchronization for 24 h with serum-deprived medium containing 5.5 mM D-glucose and 1% FBS.

To examine RAGE mRNA and protein expression in coronary artery VSMCs incubated with different concentrations of glucose, cells were cultured in 5.5, 12.0, 18.0 or 25.0 mmol/l glucose-treated medium. Following incubation for 24 or 48 h, western blot analysis and reverse-transcription quantitative polymerase chain reaction (RT-qPCR) were performed. To determine the effect of PIO, GW9662 (PPARγ antagonist), DPI (NADPH oxidase inhibitor) and PDTC (NF-κB inhibitor) on high glucose-induced RAGE expression in coronary artery VSMCs, cells were divided into the following treatment groups: i) 5.5 mmol/l D-glucose (normal glucose); ii) 25 mmol/l D-glucose (high glucose); iii) 25 mmol/l D-glucose and 10 μmol/l PIO; iv) 25 mmol/l D-glucose, 10 μmol/l PIO and 10 μmol/l GW9662; v) 25 mmol/l D-glucose and 10 μmol/l DPI and vi) 25 mmol/l D-glucose and 40 μmol/l PDTC. Cells were incubated for 48 h before western blotting and immunofluorescence to evaluate RAGE protein expression and incubated for 24 h prior to RT-qPCR analysis to evaluate RAGE mRNA expression.

The effects of PIO, GW9662, DPI and PDTC on high glucose-induced NF-κB activation and ROS production in coronary artery SMCs were evaluated. After 24 h preincubation in medium with 5.5 mmol/l D-glucose, coronary artery SMCs were exposed to the following experimental conditions for 24 h: i) 5.5 mmol/l D-glucose (normal glucose); ii) 25 mM D-glucose (high glucose); iii) 25 mmol/l D-glucose and 10 μmol/l PIO; iv) 25 mmol/l D-glucose, 10 μmol/l PIO and 10 μmol/l GW9662; v) 25 mmol/l D-glucose and 10 μmol/l DPI; vi) 25 mM D-glucose and 40 μmol/l PDTC. Following incubation, immunofluorescence was used to analyze the nuclear translocation of NF-κB and ROS levels were assessed using the CM-H2DCFDA fluoroprobe.

### RT-qPCR

Total RNA samples were extracted from rat coronary artery SMCs with TRIzol^®^ reagent (Invitrogen Life Technologies). Total RNA was reverse-transcribed with the cDNA synthesis kit according to the manufacturer’s instructions (Promega, Sunnyvale, CA, USA). qPCR was performed with 5 μl SYBR^®^ Green 2× Realtime PCR master mix, 10 μmol/l forward primer 0.2 μl, 10 μmol/l reverse primer 0.2 μl, 1 μl cDNA and 3.5 μl double-distilled H_2_0, for a total reaction volume of 10 μl. Following initial denaturation at 95°C for 3 min, PCR was performed for a total of 40 cycles, each at 95°C for 10 sec, 60°C for 10 sec and 72°C for 15 sec. The primers used were as follows: Rat-RAGE, forward 5′-AGGAGGAGACCAGGAGGCACCC-3′ and reverse 5′-CTCCCTGACTCGGGGCTGGATG-3′; rat GAPDH, forward 5′-CAAGATTGTCAGCAATGCATCC-3′ and reverse 5′-ATCACGCCACAGCTTTCCAGAG-3′. The starting copy number of the unknown samples was determined relative to the known copy number of the calibrator sample using the following formula: ^ΔΔ^Ct = [Ct target gene (unknown sample)-Ct GAPDH gene (unknown sample)]-[Ct target gene (calibrator sample)-Ct GAPDH gene (calibrator sample)]. In this case, the target gene was RAGE. The relative gene copy number was calculated by ^ΔΔ^Ct. qPCR data were normalized to an internal control (GAPDH) and were presented as the mean ± standard deviation for three independent experiments performed in triplicate.

### Western blot analysis

Cells were lysed in 1 ml cell lysate (Total Protein Extraction kit; ProMab) for 30 min at 4°C. The cell lysates were centrifuged at 12556 × g for 30 min at 4°C to remove insoluble material. The resulting supernatant was frozen at −80°C for later analyses by SDS-PAGE and immunoblotting. A total amount of 25 μl cell lysate were resolved on 12% SDS-PAGE gels (Sigma), followed by electrophoretic transfer onto polyvinylidene fluoride membranes (Pierce Biotechnology, Inc., Rockford, IL, USA). The membranes were blocked with 5% non-fat milk blocking buffer and then incubated overnight at 4°C with primary antibody (1:500 dilution; Abcam). Following extensive washing with Tris-buffered saline (TBS) containing 0.5% Tween 20 (Beyotime Institute of Biotechnology), membranes were incubated with horseradish peroxidase-labeled secondary antibody (1:4,000 dilution; Thermo Pierce, Rockford, IL, USA) at room temperature for 1 h, followed by additional washes with TBS containing 0.5% Tween 20. The blots were developed using a chemiluminescence kit. Densitometric analyses of autoradiographic bands were normalized to GAPDH expression, taking into account the size and area of the bands (Scion Image software (Scion Corp., Frederick, MD, USA).

### Immunofluorescence microscopy

Coronary artery SMCs grown on glass coverslips were washed in phosphate-buffered saline (PBS; Hyclone Laboratories, Inc.) and fixed in 4% paraformaldehyde (10 min at room temperature; Beyotime Institute of Biotechnology). Non-specific binding sites were blocked in 1% BSA (Hyclone Laboratories, Inc.) for 2 h. Cells were then incubated with a rabbit polyclonal anti-RAGE antibody (1:100 in blocking buffer) or a rabbit polyclonal anti-NF-κB p65 antibody (1:100 in blocking buffer). Following washing, cells were exposed to a goat anti-rabbit IgG-FITC (1:200) for 1 h at room temperature. Cells were mounted in 80% glycerol (Beyotime Institute of Biotechnology) and observed under a confocal microscope (LSM510; Zeiss, Oberkochen, Germany).

### Measurement of ROS levels

Coronary artery SMCs were stimulated, harvested by trypsinization, resuspended in PBS at a concentration of 10^6^ cells/ml and loaded with 10 mol/l CM-H2DCFDA (Beyotime Institute of Biotechnology). Dichlorofluorescein (DCF; Beyotime Institute of Biotechnology) fluorescence was monitored by analyzing 5,000 cells in a flow cytometer (Nikon, Tokyo, Japan).

### Statistical analysis

Data are expressed as the mean ± standard deviation. Results were analyzed using one-way analysis of variance for multiple comparisons, followed by the Fisher’s least significant difference test. Statistical analyses were performed using SPSS 16.0 software (SPSS, Inc., Chicago, IL, USA). P<0.05 was considered to indicate a statistically significant difference.

## Results

### Effect of glucose on RAGE protein and mRNA expression in coronary artery SMCs

**Figure 1:**
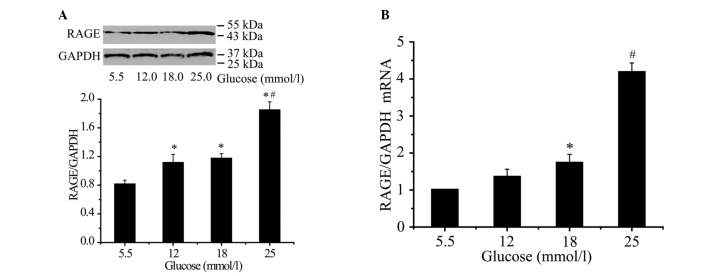
Effect of glucose on RAGE protein and mRNA expression in coronary smooth muscle cells. (A) RAGE protein expression significantly increased in cells treated with 12, 18 and 25 mmol/l glucose after a 48-h incubation. ^*^P<0.05 vs. the control group; ^#^P<0.05 vs. the 12 and 18 mmol/l glucose treatment groups. (B) RAGE mRNA expression significantly increased in 18 mmol/l (P<0.05) and 25 mmol/l (P<0.01) treatment groups after a 24-h incubation. ^*^P<0.05 and ^#^P<0.05 vs. the control group. RAGE, receptor for advanced glycation end products.

RAGE protein expression increased by 37.0, 44.4 and 127% at concentrations of 12 mmol/l (P<0.05), 18 mmol/l (P<0.05) and 25 mmol/l (P<0.01), respectively, following a 48 h incubation (Fig. 1A). RAGE mRNA expression increased by 35.1, 73.1 and 318.0% at concentrations of 12 mmol/l (P<0.05), 18 mmol/l (P<0.05) and 25 mmol/l (P<0.01), respectively, after a 24-h incubation (Fig. 1A). The effect of glucose on RAGE mRNA and protein expression was concentration dependent. Maximal glucose treatment was reached at 25 mmol/l (P<0.01; Fig. 1B).

### PIO, DPI and PDTC decreases high glucose-induced RAGE expression of coronary artery VSMCs

**Figure 2:**
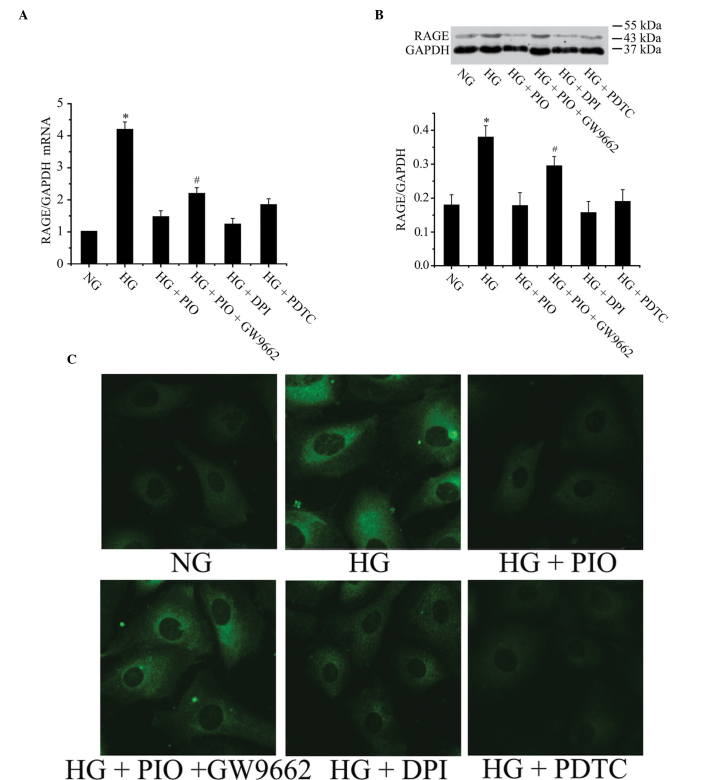
PIO, DPI and PDTC decrease HG-induced RAGE expression in coronary vascular smooth muscle cells. (A) RAGE mRNA declined significantly following pretreatment with PIO, DPI or PDTC, respectively. Pretreatment of PIO plus GW9662 induced increased RAGE mRNA expression compared with treatment only by PIO. ^*^P<0.05 vs. the NG, HG+PIO, HG+DPI or HG+PDTC groups; ^#^P<0.05 vs. the HG+PIO group. (B) PIO, DPI and PDTC significantly inhibited HG-stimulated RAGE expression. Pretreatment of PIO plus GW9662 induced increased expression level of RAGE compared with treatment of only PIO. ^*^P<0.05 vs. other groups; ^#^P<0.05 vs. HG+PIO group. (C) RAGE associated immunofluorescence result was in agreement with previous observations of RAGE expression as assessed by western blot analysis (magnification, ×400). NG, normal glucose; HG, high glucose; PIO, pioglitazone; PDTC, pyrrolidine dithiocarbamate; DPI, diphenyleneiodonium; RAGE, receptor for advanced glycation end products; NG, normal glucose; HG, high glucose.

It was identified that 10 μmol/l PIO (PPARγ agonist), 10 μmol/l DPI (NADPH oxidase inhibitor) and 40 μmol/l PDTC (NF-κB inhibitor) significantly inhibited high glucose-stimulated RAGE expression. RAGE mRNA expression declined by 65.2, 70.7 and 56.1% following pretreatment with PIO, DPI or PDTC respectively (Fig. 2A). RAGE protein expression decreased by 52.8, 58.9 and 50.1% following pretreatment with PIO, DPI, or PDTC, respectively (Fig. 2B). These findings suggest that high glucose-induced RAGE expression is dependent upon activation of NADPH oxidase, ROS generation and NF-κB activation. PIO may mimic the effect of DPI or PDTC, significantly downregulating high glucose-stimulated RAGE expression. Pretreatment of PIO with GW9662 induced the lower expression of RAGE compared with treatment with PIO only, which indicates that PIO inhibits RAGE expression through interaction with PPARγ. The immunofluorescence results were in accordance with our previous assessment of RAGE protein expression by western blot analysis (Fig. 2C).

### PIO, DPI and PDTC decreases high D-glucose-induced NF-κB activation of coronary artery SMCs

**Figure 3:**
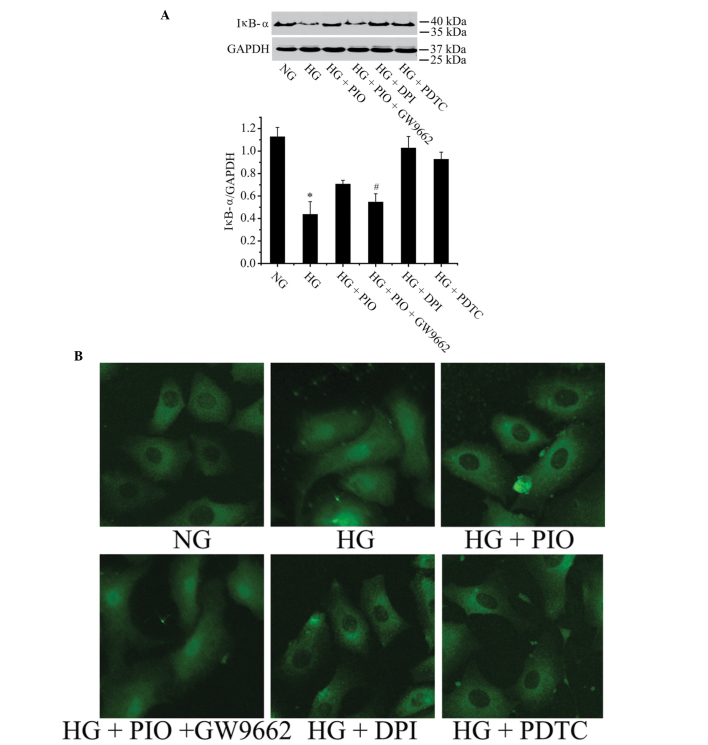
Peroxisome proliferator-activated receptor γ agonist PIO, nicotinamide adenine dinucleotide phosphate oxidase inhibitor DPI and NF-κB inhibitor PDTC decrease high D-glucose-induced NF-κB activation of coronary artery smooth muscle cells. (A) HG treatment significantly decreased cytoplasmic I-κBα protein levels. PIO, DPI or PDTC were able to inhibit this effect. Furthermore, addition of GW9662 eliminated the inhibitory effect of PIO. ^*^P<0.05 vs. the other groups; ^#^P<0.05 vs. the HG+PIO group. (B) PIO inhibited HG-induced NF-κB translocation and the inhibition was eradicated by GW9662. DPI and PDTC may also inhibit HG-induced NF-κB translocation (magnification, ×400). NG, normal glucose; HG, high glucose; PIO, pioglitazone; PDTC, pyrrolidine dithiocarbamate; DPI, diphenyleneiodonium; RAGE, receptor for advanced glycation end products; NG, normal glucose; HG, high glucose; NF-κB, nuclear factor-κB; I-κBα, NF-κB inhibitor α.

The mechanism by which PIO inhibited high glucose-induced RAGE expression in coronary artery VSMCs was further examined. It has been observed that the RAGE promoter possesses NF-κB binding sites. It was therefore evaluated whether PIO functioned by affecting the NF-κB pathway. High glucose treatment significantly decreased cytoplasmic IκBα protein levels. When PIO was included in the culture medium, this effect was inhibited. Furthermore, addition of GW9662 eradicated the inhibitory effect of PIO (Fig. 3A). Immunofluorescence was used to detect the nuclear translocation of NF-κB in coronary artery VSMCs cells in response to high glucose (Fig. 3B). NF-κB primarily existed in the cytoplasm of control cells, but translocated from the cytoplasm to the nucleus following treatment with 25 mmol/l glucose. However, PIO inhibited high glucose-induced NF-κB translocation and this inhibition was eradicated with GW9662 (Fig. 3B). DPI and PDTC were also able to inhibit high glucose-induced NF-κB translocation. Taken together, the present findings demonstrate that PIO may inhibit NF-κB signaling through the PPARγ receptor in response to high glucose levels, which partially explains the inhibitory effect of PIO on high glucose-induced RAGE expression in coronary artery VSMCs.

### PIO and DPI decrease high D-glucose-induced ROS production

**Figure 4:**
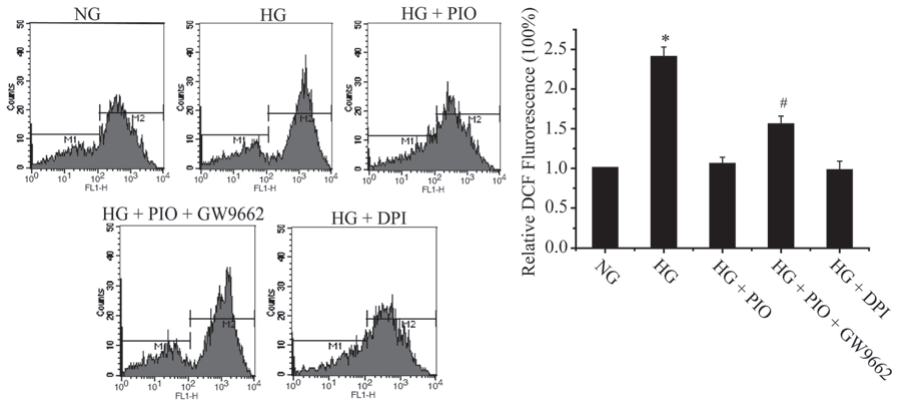
Peroxisome proliferator-activated receptor γ agonist PIO and nicotinamide adenine dinucleotide phosphate oxidase inhibitor DPI decreases high D-glucose-induced ROS production. ROS levels in coronary artery smooth muscle cells were assessed by DCF fluorescence by flow cytometry. Significantly increased levels of ROS compared with control cells were observed following treatment with HG. Pretreatment with DPI (10 μmol/l) or PIO for 30 min significantly decreased HG-induced ROS elevation. ^*^P<0.05 vs. other groups; ^#^P<0.05 vs. the HG+PIO group. NG, normal glucose; HG, high glucose; PIO, pioglitazone; DPI, diphenyleneiodonium; ROS, reactive oxygen species; DCF, dichlorofluorescein.

To investigate whether PIO may decrease ROS levels, coronary artery SMCs were treated with high glucose (25 mmol/l) for 24 h and ROS levels were measured by DCF fluorescence in a flow cytometer. Significantly increased levels of ROS compared with control cells were observed following treatment with high glucose. Pretreatment with DPI or PIO significantly decreased high glucose-induced ROS elevation, as measured by DCF fluorescence (Fig. 4).

## Discussion

A large body of experimental evidence supports the integral contribution of RAGE activation to all major stages associated with the development and progression of atherosclerosis in diabetes. RAGE activation was able to elicit ROS generation and subsequently induce SMC proliferation (10,11). The source of RAGE activation mediated ROS production is supposedly NADPH oxidase and in cultured SMCs, various downstream signaling pathways, including NF-κB, are activated by stimulation of RAGE (12). AGEs stimulate collagen synthesis activity and induce cell-associated fibronectin production and TGF-β expression in cultured SMCs through interaction with RAGE (13). The AGE-RAGE interaction stimulates expression of typical bone proteins, including alkaline phosphatase, osteopontin and osetocalcin in cultured SMCs and therefore increases calcification in the arteries (14–16).

It has been demonstrated that the expression of RAGE in coronary arterioles was markedly increased in diabetic vs. control mice, but the underlying mechanism remains to be elucidated. One of the major findings of the present study is that glucose was able to directly induce RAGE protein and mRNA expression of coronary artery SMCs in a concentration-dependent manner, which is one of the mechanisms underlying elevated RAGE expression in the coronary arteries of diabetic mice. Former studies revealed that in diabetic RAGE^−/−^/apolipoprotein E (ApoE)^−/−^ double knockout mice, the absence of RAGE was associated with the significant attenuation of atherosclerotic plaque accumulation. Overexpression of RAGE induces the expression of a number of proinflammatory genes (MCP-1, IL-6 and ICAM-1) in VSMCs (17). Overexpression of RAGE in coronary artery VSMCs induced by high glucose may be critical in accelerating inflammation and atherosclerosis of coronary arteries.

In diabetic RAGE^−/−^/ApoE^−/−^ double knockout mice, expression of the aortic NF-κB p65 subunit, inflammatory cytokines, adhesion molecules, including VCAM-1, MCP-1 and certain NADPH oxidase subunits, including gp91phox, p47phox and rac-1 were decreased significantly when compared with diabetic ApoE^−/−^ mice (18–20). These studies not only illustrated the importance of RAGE in atherosclerosis progression in diabetic mice, but also indicated that RAGE activation is closely associated with the NF-κB pathway and ROS originating from NAPDH oxidase. The association between increased expression of RAGE and elevated ROS production as well as NF-κB activation remains to be investigated further.

In the present study, the mechanism underlying high glucose-induced RAGE expression was investigated. In accordance with former studies, high glucose was able to induce ROS production following treatment for 12 h. Following inhibition of NADPH oxidase by DPI, high glucose-induced ROS production and RAGE expression in coronary artery SMCs significantly decreased compared with the high glucose treatment group. Therefore, it is hypothesized that high glucose elicits the generation of ROS and subsequently induces RAGE expression in coronary artery SMCs. Previous studies have demonstrated that the RAGE promoter possesses NF-κB binding sites (21). In the present study, NF-κB activation was significantly inhibited by PDTC and DPI; inhibition of NF-κB activation by PDTC was able to downregulate high glucose-induced RAGE expression. Thus, it is hypothesized that high glucose upregulates RAGE expression by stimulating ROS production and activating the NF-κB signaling pathway.

PIO is an anti-diabetic insulin-sensitizing agent that improves insulin action in a variety of animal models of insulin resistance and diabetes. It is considered to exert such effects by acting as a selective ligand of PPARγ receptors. In addition, previous studies have demonstrated that PIO not only ameliorates insulin sensitivity, but also has anti-inflammatory, antioxidative and antiproliferative effects on vascular wall cells. It has been demonstrated that ligands of PPARγ receptors, including PIO inhibit VSMC ROS production and inflammation, which are important in the process of atherosclerosis formation in diabetes. Previous studies have revealed that PIO inhibits AGE-induced VSMC proliferation via increasing PPARγ expression and inhibiting the ROS/ERK1/2 signaling pathway (22). In the current study, it was observed that PIO decreased high glucose-induced upregulation of RAGE expression in coronary artery VSMCs, whereas pretreatment of SMCs with PIO and GW9662 did not downregulate RAGE expression. These findings raised the possibility that PIO may decrease RAGE expression in coronary artery SMCs through a PPARγ-dependent mechanism. The mechanism of RAGE downregulation by a PPARγ agonist was also investigated. In cultured coronary artery SMCs, it was observed that high glucose potently induced intracellular ROS production. DPI pretreatment significantly attenuated RAGE expression induced by high glucose by scavenging ROS production. Additionally, PIO was able to mimic the effects of DPI in coronary artery SMCs. These results suggest that alleviating oxidative stress using PIO is able to inhibit RAGE expression in coronary artery SMCs.

In the present study, it was observed that PDTC, DPI and PIO inhibited NF-κB activation in high glucose-treated VSMCs. Furthermore, pharmacological inhibition of NF-κB activation by PDTC or treatment with PIO significantly downregulated high glucose-induced RAGE expression in coronary artery SMCs. Taken together, these results suggest that PIO inhibits high glucose-induced RAGE expression through inhibiting ROS-mediated NF-κB activation. In conclusion, the present study demonstrated that high glucose may directly induce RAGE expression through stimulating ROS-mediated NF-κB activation, which may be inhibited by PIO. These data provide novel insights into the molecular pathways underlying increased RAGE expression in the coronary artery SMCs stimulated by high glucose, and reveal novel mechanisms that contribute to the beneficial effects of PIO in coronary atherosclerosis in diabetes.
